# Effects of Exercise Domain and Intensity on Sleep in Women and Men with Overweight and Obesity

**DOI:** 10.1155/2019/2189034

**Published:** 2019-04-07

**Authors:** Jonas Salling Quist, Mads Rosenkilde, Anne Sofie Gram, Martin Bæk Blond, Daniel Holm-Petersen, Mads Fiil Hjorth, Bente Stallknecht, Anders Sjödin

**Affiliations:** ^1^Department of Biomedical Sciences, Faculty of Health and Medical Sciences, University of Copenhagen, Copenhagen, Denmark; ^2^Department of Nutrition, Exercise and Sports, Faculty of Science, University of Copenhagen, Copenhagen, Denmark

## Abstract

Inadequate sleep is associated with cardiometabolic risk and adiposity. Exercise has been suggested as an efficient strategy to improve sleep; however, the effects of different types of exercise on sleep in individuals with overweight and obesity are not well understood. We examined effects of active commuting and leisure-time exercise on sleep in individuals with overweight or obesity. 130 physically inactive adults (20–45 years) with overweight or class 1 obesity (body mass index: 25–35 kg/m^2^) were randomized to 6 months of habitual lifestyle (CON, *n* = 18), active commuting by bike (BIKE, *n* = 35), or leisure-time exercise of moderate intensity (MOD, 50% VO_2_peak-reserve, *n* = 39) or vigorous intensity (VIG, 70% VO_2_peak-reserve, *n* = 38), 5 days/week. Sleep was assessed from 7-day/night accelerometry and questionnaires at baseline, 3 months, and 6 months. 92 participants were included in a *per protocol* analysis. At 3 months, sleep duration was longer in VIG (29 min/night [3; 55] (mean [95% CI]), *p*=0.03) but not in BIKE and MOD (*p* ≥ 0.11) compared with CON and was not different between groups at 6 months (*p* ≥ 0.36 vs. CON). At 6 months, sleep duration variability was lower in MOD (−31% [−50; −3], *p*=0.03) and numerically lower in VIG (−28% [−49; 1], *p*=0.06) relative to CON but was unchanged in BIKE (*p*=0.17 vs. CON). The effects were, however, primarily attributable to shorter and more irregular sleep in CON over time. Our findings suggest that effects of exercise on sleep in individuals with overweight and obesity may be restricted to leisure-time exercise with a short-term effect on sleep duration after vigorous intensity exercise (3 months) but a more regular sleep pattern after 6 months of moderate and vigorous intensity exercise compared with physically inactive controls. This trial was registered at clinicaltrials.gov with ID NCT01962259.

## 1. Introduction

Poor sleep and sleep deprivation are common phenomena in modern 24 h societies [1]. A growing body of evidence suggests that short sleep duration, poor sleep quality, and irregular sleep patterns are associated with increased risk of obesity and the metabolic syndrome [1]. Potential mechanisms linking poor sleep to obesity and cardiometabolic risk include upregulation of appetite and reduced motivation to be physically active [2, 3].

Regular exercise is generally believed to improve sleep in individuals with sleep complaints or sleep disorders as well as among healthy individuals [4, 5], and physical activity has been associated with a lower risk of daytime sleepiness [6] and sleep problems [7–9]. It has been suggested that both acute and regular exercise may improve a variety of sleep outcomes including sleep duration and subjective sleep quality [10, 11]. In a previous pilot study, we observed increased objectively measured sleep duration with a high daily amount (60 min), but not a moderate amount (30 min) of vigorous intensity exercise for 3 months in men with overweight, suggesting a certain amount of exercise is needed to improve sleep [12]. Furthermore, it has been suggested that exercise has to be of vigorous intensity in order to induce beneficial effects on sleep outcomes in younger populations [13].

The effects of different types of exercise on sleep are not well investigated, and the majority of previous studies investigating effects of exercise on sleep have been conducted in leisure-time. Active commuting is a promising time-efficient alternative to leisure-time exercise, but whether active commuting exerts beneficial effects on sleep comparable to those of leisure-time exercise has not been investigated. The aim of the present study was to investigate the effects of 6 months of active commuting and leisure-time exercise of moderate or vigorous intensity on objectively measured sleep duration and patterns as well as subjectively rated sleep quality and sleepiness in individuals with overweight and obesity. Additionally, we aimed to investigate potential associations between changes in sleep and adiposity during the intervention.

## 2. Methods

The data reported here constitute a part of a larger interdisciplinary research project, Governing Obesity—Active Commuting To Improve health and Wellbeing of Everyday life (GO-ACTIWE), investigating the health effects of different types of exercise in adults with overweight and class 1 obesity. The GO-ACTIWE trial was a 6-month randomized controlled trial, and the study design has been described in detail elsewhere [14].

The results on fat loss [15], appetite [16], low-grade inflammation and endothelial function [17], and fibrin turnover [18] in GO-ACTIWE have been reported elsewhere. We followed the methods previously described in details in Rosenkilde et al. [14] and Quist et al. [15], but in order to facilitate reading, we present a detailed description of the methodology relevant for the present analysis. The study was performed at the Department of Biomedical Sciences, Faculty of Health and Medical Sciences, University of Copenhagen, Denmark, between November 2013 and June 2016.

### 2.1. Participants

We recruited healthy, physically inactive, Caucasian women and men aged 20–45 years with overweight or class 1 obesity (body mass index: 25–35 kg/m^2^). The exclusion criteria were unstable body weight (>±4 kg) within the last 6 months, body fat <32% for women and <25% for men, physically active (structured exercise >2 times/week or bicycling >25 km/week), peak oxygen uptake (VO_2_peak) >40 ml O_2_/kg/min for women and >45 ml O_2_/kg/min for men, blood pressure >140/90 mmHg, abnormal rest or working electrocardiography or regular use of medication, smoking, fasting blood glucose >6.1 mmol/l, and first-degree relatives with type 2 diabetes and for women, menopause and postmenopausal status and pregnancy or planning of pregnancy within the following year. Participants engaged in shift work were excluded from the present analyses. The rationale for inclusion of younger apparently healthy adults with a physically inactive lifestyle and with overweight or obesity is that this phenotype is highly prevalent in the general population and is considered at increased risk of lifestyle-related diseases. From a preventive public health perspective, studies targeting this group of individuals are relevant.

Eligible participants received oral and written information and signed informed consent prior to attending a screening visit which included medical history and assessment of the abovementioned criteria. The study was approved by the ethical committee of the Capital Region of Denmark (H-4-2013-108), registered at the Danish Data Protection Agency and at clinicaltrials.gov (ID: NCT01962259) and was conducted in accordance with the Declaration of Helsinki.

### 2.2. Study Design and Randomization

The original study design included a 12-month intervention, but the duration was shortened to 6 months (February 2, 2014) without reference to data owing to an unexpectedly large withdrawal of eligible individuals during the inclusion phase compared to our previous studies of shorter duration [19, 20]. After baseline testing, participants were randomly allocated in a gender-stratified 1 : 2 : 2 : 2 manner using a permuted block design of 7–14 participants per strata to control (CON, *n* = 18), active commuting by bike (BIKE, *n* = 35), or leisure-time exercise of moderate intensity (MOD, *n* = 39) or vigorous intensity (VIG, *n* = 38). A lower number of participants were allocated to CON because of ethical considerations associated with maintenance of a physically inactive lifestyle throughout the study. Four participants (MOD, *n* = 3; VIG, *n* = 1) engaged in shift work were excluded from the present analyses. Dietary intake was *ad libitum* but participants were advised to continue habitual eating pattern throughout the study. No instructions regarding sleep were given to the participants.

### 2.3. Exercise Interventions

Exercise was prescribed in addition to participants' habitual physical activity, including potential active commuting or exercise sessions, and participants were instructed to maintain their habitual physical activities throughout the intervention. After a 3-week ramp-up period, participants were instructed to exercise 5 days/week with exercise energy expenditure at 320 kcal/day for women and 420 kcal/day for men, equilibrating exercise energy expenditure across genders to approximately 33 kcal/kg fat free mass/week [14], which are in line with most health recommendations [21, 22]. In BIKE, the intensity was self-chosen and participants were instructed to commute by bike back and forth from work or school, and an individual average daily distance was calculated to achieve the same exercise energy expenditure as the other intervention groups. Furthermore, participants in BIKE that did not own a bike suitable for the intervention were provided a bike (Nishiki Touring Master, 7 speeds, Nishiki bikes, Gothenburg, Sweden). MOD and VIG had free access to a fitness center and were instructed to perform aerobic exercise (e.g., walking, running, rowing, or cycling). In MOD and VIG, exercise intensity was prescribed at 50% and 70% VO_2_peak-reserve, respectively, and target exercise heart rate was adjusted during the intervention at 1.5 and 3 months. A heart rate monitor with GPS (Polar RC3 GPS, Polar Electro Oy, Kempele, Finland) was worn during all exercise/commuting sessions in order to monitor compliance, and participants were instructed to upload exercise heart rate data every week. We aimed to keep weekly compliance between 80 and 120% of exercise prescription, and we were in weekly contact with the participants. In case of deviations from the prescribed exercise, participants were asked to increase or reduce daily exercise energy expenditure by 25% until the participant was within the range again.

### 2.4. Tests and Measurements

Three identical test periods were scheduled at baseline, 3 months, and 6 months. Participants were asked to follow their habitual lifestyle and randomization (3 and 6 months testing) during the test periods but no exercise or alcohol consumption was allowed the day before a test day.

### 2.5. Objective Assessment of Sleep

Sleep duration was assessed from accelerometry (Actigraph GT3x, Actigraph Corp., Pensacola, Florida, USA) for 8 nights. Measurements were initiated during a test day including meal and exercise challenges [16], and the first night was omitted to avoid potential influence of these stimuli on subsequent sleep, leaving 7 nights for analysis. Participants were instructed to wear the accelerometer at the waist for 24 h/day and were only allowed to take it off during water activities. Acceleration data were sampled at 30 Hz and downloaded as 60 sec epochs (ActiLife6, ver. 6.11.4, Actigraph Corp., Pensacola, Florida, USA). Measurements were considered valid if sleep data were available for ≥4 days including a minimum of 1 weekend day. Along with the measurements, participants were asked to keep a sleep log including time to bed, time to sleep, and wake-up time. Self-reported time to sleep and wake-up time were used as the possible window of sleep, and sleep was scored using the algorithm by Cole et al. [23]. Nights with zero activity were considered as nonwear time and excluded from the analyses. Sleep logs were missing from eight assessment periods from six participants included in the present analysis. In these cases, possible windows for sleep were visually identified from the individual actograms. Data from four participants at four assessment periods (3 months: CON, *n* = 1; MOD, *n* = 2; 6 months: BIKE, *n* = 1) were omitted from the present analysis due to illness during measurements.

Accelerometer estimated sleep duration is longer when assessed from a device worn at the waist compared to on the wrist [24]. However, we have now reanalyzed data from our previous study in children [24] and found no difference between changes in sleep duration as assessed by Actigraph GT3x worn at the waist and wrist, respectively (−1.5 min/night [95% CI: −6.3 to 3.2], *p*=0.52) (Supplementary Table 1). In addition, the changes in sleep duration between attachment sites were strongly correlated (*r* = 0.83, *p* < 0.001) (Supplementary Table 1 and Supplementary Figure 1). Therefore, the bias associated with accelerometer attachment site is not expected to affect the findings on changes in sleep duration in the present intervention study. The validity of waist-worn accelerometers to measure sleep efficiency and wakefulness can be questioned [24], owing to possible insufficient sensitivity to detect limited movements of the trunk during sleep. Therefore, we do not report data on sleep efficiency and wakefulness in the present analysis.

#### 2.5.1. Sleep Parameters and Calculations

*Sleep duration* (min/night) is presented as the average during the entire week but also as the average during weekdays and weekend days separately. In order to assess regularity of sleep during the week, *sleep duration variability* (min/night) was calculated as the sum of the absolute differences between the mean sleep duration and sleep duration at each night divided by the number of nights measured [25]. In order to assess fluctuations in sleep timing during the week, independent of sleep quantity, *sleep midpoint variability* (min/night) was calculated as the sum of the absolute differences between the mean sleep midpoint and sleep midpoint during each night divided by the number of nights measured.

### 2.6. Assessment of Sleep Quality and Daytime Sleepiness

*Sleep quality* was assessed using the Pittsburgh Sleep Quality Index (PSQI) [26]. The questionnaire covers sleep quality and sleep disturbances over the preceding month and includes 19 individual items that generate seven scores weighted on a 0–3 scale. The sum of the component scores yields a total score of 0–21, and the higher the score, the poorer the sleep quality. A total score >5 has been suggested as a sensitive measure of poor sleep quality [26].

*Sleepiness* was assessed by the Epworth Sleepiness Scale which is a questionnaire developed to assess daytime sleepiness in adults [27]. The questionnaire contact information and permission to use Mapi Research Trust, Lyon, France–Internet: https://eprovide.mapi-trust.org. The questionnaire describes eight different situations of everyday life, and participants were asked to rate how likely they would doze off or fall asleep in each situation on a 0–3 scale. The sum of the scores yields a total score of 0–24, with higher scores indicative of greater daytime sleepiness [27].

### 2.7. Assessment of Anthropometry and Cardiorespiratory Fitness

*Anthropometry* was assessed after an overnight fast (≥10 h). Body weight and height were measured using a combined scale and stadiometer (SECA 767, Vogel&Halke, Hamburg, Germany). Body composition was measured using dual-energy X-ray absorptiometry (DPX-IQ X-ray bone densitometer 4.7e, Lunar Corporation, Madison, WI, USA). Waist circumference (WC) was measured after mild expiration around the narrowest part between the lowest rib and the iliac crest.

*Cardiorespiratory fitness* was determined as VO_2_peak measured by indirect calorimetry (Oxycon Pro, Jaeger, Würzburg, Germany) during a graded bicycle test on an electronically braked stationary bicycle (Lode Excalibur, Groningen, Netherlands).

### 2.8. Statistics

The present study aimed to assess the efficacy of the different exercise interventions on sleep parameters and was therefore performed as *per protocol* analysis including participants who adhered to the intervention and completed testing at 3 and/or 6 months. Baseline characteristics of completers and noncompleters were compared using a *t*-test. Training compliance was compared between groups using 1-way ANOVA, and in case of a significant *F*-test, post hoc *t*-tests were performed to compare individual groups. Intervention effects were calculated as response profiles representing differences between groups for participants who completed testing at 3 and 6 months, respectively. Changes after 3 and 6 months were evaluated using a mixed model with mean value as a function of time and group by time interaction adjusted for gender, age, and baseline variation. Baseline variation was adjusted for by taking the correlation between repeated measures on the individual level into account. An unstructured covariance was used to model the association between the three measurements (baseline, 3 months, and 6 months) on each participant. Multiple regression analyses were performed to assess potential associations between changes in sleep and adiposity including participants from the intervention groups and were adjusted for age, gender, and exercise intervention group as well as the specific sleep and adiposity parameters at baseline. Raw descriptive data are presented as unadjusted mean (standard deviation) for parametric data and as median (interquartile range) for nonparametric data (Tables 1 [Table tab2]–3). Nonparametric data were logarithmically transformed. Estimated changes and differences are presented as mean (95% confidence intervals) for parametric data and as percentage (95% confidence intervals) for nonparametric data (Table 4). The primary objective was to compare effects in the intervention groups with the control group which was instructed to maintain their habitual lifestyle. Therefore, results are effect sizes of comparisons with the control group (Table 4). Additionally, we tested potential differences between the three exercise arms (Table 4). Adequacy of the assumptions of normality and homogeneity of variances were assessed using graphical methods. Corrections for multiple comparisons were not performed. A *p* value < 0.05 (two-tailed) was considered significant, and statistical analyses were performed in SAS Enterprise Guide 7.1 (SAS Institute, Cary, NC, USA).

## 3. Results

### 3.1. Participants and Training Compliance

Baseline characteristics are presented in Tables 1 and 3. There were no significant differences between participants who completed 3 and/or 6 months intervention and noncompleters (*p* ≥ 0.10 for all comparisons). Number of participants with valid sleep data at baseline, 3 months, and 6 months is presented in Table 3. Training compliance is presented in Table 2. Participants in all three exercise groups demonstrated excellent adherence to the prescribed exercise energy expenditure (≥90%). As per study design, exercise intensity (%VO_2_peak-reserve) was moderate in MOD (49%) and vigorous in VIG (65%), and therefore, exercise intensity deviated slightly from prescribed in VIG (70% VO_2_peak-reserve) over the entire course of the intervention. The self-selected intensity was moderate (54%) in BIKE. Duration of exercise sessions was on average 46 min in BIKE, 54 min in MOD, and 37 min in VIG. Training compliance in GO-ACTIWE including a slightly different number of participants has been reported elsewhere [15–18]. 95% of all accelerometer measurements covered 6–7 days and nights (Table 3).

### 3.2. Sleep Duration and Variability in Sleep Duration

At 3 months, sleep duration was longer in VIG compared to CON (*p*=0.03), which was primarily attributable to a reduction in CON, whereas sleep duration did not differ in BIKE and MOD compared with CON (both *p* ≥ 0.11) (Table 4). Moreover, sleep duration was longer in VIG compared to BIKE after 3 months (27 min/night [4; 49], *p*=0.02), which was also primarily due to a decrease in sleep duration in BIKE (Table 4). No changes in sleep duration were, however, observed in any of the intervention groups after 6 months (*p* ≥ 0.36 vs. CON) (Table 4). Sleep duration variability was not changed in any of the intervention groups after 3 months (*p* ≥ 0.35 vs. CON) (Table 4). However, after 6 months, sleep duration variability was lower in MOD (*p*=0.03) and numerically lower in VIG (*p*=0.06) compared with CON, whereas no difference was observed between BIKE and CON (*p*=0.17) (Table 4). The effects were, however, primarily attributable to more irregular sleep in CON over time. Additional adjustment for season did not change the results on sleep duration and sleep duration variability (data not shown).

Sleep duration during weekdays was longer after 3 months in MOD (*p*=0.03) and VIG (*p*=0.047) but not in BIKE (*p*=0.52) compared with CON, whereas no differences were observed after 6 months (*p* ≥ 0.69 vs. CON) (Table 4). Weekend sleep duration did not differ in any of the exercise groups compared with CON after 3 and 6 months (*p* ≥ 0.15) but was longer in VIG after 3 months compared to BIKE (*p*=0.02) and MOD (*p*=0.04) (Table 4). Sleep midpoint variability was unaltered in all of the exercise groups after 3 and 6 months (*p* ≥ 0.13 vs. CON) (Table 4).

### 3.3. Sleep Quality and Sleepiness

Out of the 90 participants who adhered to the intervention and from whom valid questionnaire data were available at 3 and/or 6 months, 38 participants (42%) (CON: *n* = 6 (38%); BIKE: *n* = 7 (35%); MOD: *n* = 13 (43%); VIG: *n* = 12 (50%)) had poor self-reported sleep quality (PSQI total score >5) at baseline. Except for a within-group improvement in self-reported sleep quality in VIG after 3 months, which was not different from CON (*p*=0.57), and a numerical reduction in sleepiness in BIKE compared with CON after 6 months (*p*=0.08), no changes in reported sleep quality or sleepiness were observed in any of the groups after 3 or 6 months compared with CON (Table 4).

### 3.4. Cardiorespiratory Fitness and Body Composition

Changes in VO_2_peak and body composition in GO-ACTIWE have been reported elsewhere [15–18]. For the participants included in the present analysis, VO_2_peak increased in all three intervention groups at 6 months (*p* ≤ 0.01 vs. CON), while body weight and body fat percentage decreased (*p* ≤ 0.02 vs. CON) (data not shown).

### 3.5. Associations between Changes in Sleep and Changes in Adiposity

Improved self-reported sleep quality was associated with reductions in fat percentage (*p*=0.03) and WC (*p*=0.02) during the first 3 months but not during all 6 months (*p* ≥ 0.66) (Table 5). Moreover, decreased self-reported sleepiness was similarly associated with reductions in fat percentage from baseline to 3 months (*p*=0.01) and with reductions in android fat percentage from baseline to 3 and 6 months (*p* < 0.001 and *p*=0.03, respectively) (Table 5). Increased fluctuations in sleep midpoint were associated with reductions in android fat percentage during the first 3 months (*p*=0.03); however, no other significant associations between changes in accelerometer-determined sleep and changes in adiposity were observed (*p* ≥ 0.07) (Table 5).

## 4. Discussion

This is the first randomized controlled trial reporting effects of active commuting as well as leisure-time exercise of different intensities on sleep in individuals with overweight or moderate obesity. We observed that sleep duration was ∼30 min/night longer after 3 months of vigorous intensity exercise compared to controls. This effect, however, was not sustained after 6 months, suggesting only a short-term effect of exercise intensity on sleep duration. The effects on sleep duration variability were not seen until after 6 months of moderate leisure-time exercise where sleep duration variability was also numerically lower after vigorous intensity exercise compared to controls. No effects on sleep were observed after active commuting.

Previous intervention studies have generally reported small but beneficial effects of regular exercise on sleep duration [10]. Studies have, however, primarily investigated the effects of moderate intensity exercise and the combination of exercise and other behavioral interventions which complicates comparison of findings between studies [5, 10]. In our previous pilot study, a within-group increase in accelerometer assessed sleep duration was observed after a high (∼60 min) but not a moderate (∼30 min) daily dose of vigorous intensity leisure-time exercise for 3 months in a small number of younger overweight men [12]. A within-group increase in accelerometer determined sleep duration has also been reported in adolescents with obesity after 12 weeks of combined moderate-to-vigorous intensity exercise and resistance training [28]. However, these changes were not significantly different from controls and should therefore be interpreted with caution.

Our findings suggest that almost similar effects on sleep duration variability can be obtained regardless of the intensity of exercise, but that the effect of exercise on sleep duration seems to be dependent on exercise intensity. Observations from short-term (<1 week) studies suggest that similar effects on sleep outcomes can be obtained across light, moderate, and vigorous intensity exercise [10]. The attenuation of the effects on sleep duration seen after 6 months is in line with previous studies suggesting a duration-dependent attenuation of the effect of exercise on sleep duration [10]. This could possibly be related to adaptations to the exercise stimulus, although the longer sleep duration in the vigorous intensity exercise group compared with controls at 3 months was primarily attributable to a reduction in sleep duration within the control group. Short sleep duration and poor sleep quality are common among elite athletes [29, 30], and we cannot exclude the existence of a given threshold above which exercise exerts adverse effects on sleep. Furthermore, it has been proposed that day-time napping may be present among athletes involved in individual sports [29]. Nocturnal sleep was the focus in the present study, but some participants anecdotally reported day-time napping. Day-time napping could potentially affect night-time sleep duration and *vice versa*; however, this remains speculation since napping was not systematically reported. Seasonal variation in sleep duration has been reported [31, 32]; however, our results on sleep duration and sleep duration variability did not change when additionally adjusted for season.

We and others have previously observed positive associations between sleep duration variability and intake of sugar-sweetened beverages [25] as well as abdominal adiposity [33]. The lower sleep duration variability observed in response to moderate intensity exercise and the numerically lower sleep duration variability after vigorous intensity exercise after 6 months are considered clinically relevant due to the adverse effects of circadian misalignment of sleep on cardiometabolic risk [1, 34].

It remains unclear why participants did not benefit from active commuting with regards to sleep outcomes. However, it may possibly be explained by the greater than expected drop-out rate in BIKE and thereby lack of statistical power. In BIKE, daily exercise was usually obtained from two separate sessions, which could possibly induce less fatigue compared to MOD and VIG. Although speculative, logistics related to implementation of exercise in everyday life may have affected participants' sleep pattern. If commuting time was increased in BIKE, this may have affected sleep duration, as suggested by cross-sectional associations between longer commuting time and shorter sleep duration [35]; however, sleep duration was only reduced within BIKE during the first 3 months of the intervention. We have no reasonable explanation for the reduction in sleep duration in CON during the first three months of the intervention.

Compliance to the prescribed exercise was excellent in all three intervention groups. According to questionnaire data, weather was identified to have a significant impact on motivation to perform outdoor exercise (unpublished data). Since participants were recruited continuously, it cannot be excluded that season and weather might have affected motivation to exercise which may have been especially pronounced in BIKE; however, the relatively mild winter in Denmark allows for bicycling all year. The study was conducted in the greater Copenhagen area, which is a bike-friendly environment. This may limit the generalizability to other cities.

Except for a within-group improvement in self-reported sleep quality in VIG after 3 months, which was not different from CON, we did not observe changes in self-reported sleep quality. Improvements in subjective sleep quality have been reported after 6 months of different doses of exercise in postmenopausal women with overweight and obesity [36] and in several studies in middle-aged and older individuals with sleep complaints after different exercise prescriptions [10, 13, 37]. The most obvious explanation for the lack of change in some of the sleep outcomes assessed is the inclusion of healthy participants with good sleep to the study. We did not specifically recruit participants based on their sleep pattern and a potential ceiling effect may explain the lack of changes [38]. Inclusion of individuals with greater degrees of adiposity and poor sleep could likely have resulted in greater effects on sleep outcomes.

Although the causality remains uncertain and effect sizes are small, improvements in self-reported sleep quality were associated with greater reductions in body fat and WC during the first 3 months, which may be explained by most fat being lost during this period [15]. Furthermore, decreased sleepiness was associated with reductions in android fat after 3 and 6 months. The latter observations are in line with previous studies reporting associations between improvements in sleepiness and reductions in weight and WC during weight loss interventions [39]. Participants who felt less sleepy in response to the intervention could very likely be more physically active and spend less time on sedentary activities that are also associated with consumption of energy-dense snacks and beverages [40]. Furthermore, overweight and obesity are associated with sleep apnea [41]. Although we did not assess sleep apnea in the present study, exercise and weight loss may have resulted in reductions in sleep apnea [42, 43].

A number of limitations could influence the interpretation of our observations. The validity and reliability of wrist-worn accelerometers for assessment of sleep are considered reasonable [44], but waist-worn accelerometers are known to overestimate sleep duration compared to wrist-worn accelerometers, and assessment of sleep efficiency and wakefulness is probably questionable [24]. Therefore, we did not include sleep efficiency and wakefulness in the present analysis and are not able to determine effects of exercise on objective measures of sleep quality. Waist was used as attachment site in the present study in order to assess physical activity and sleep using a single accelerometer and to minimize risk of potential compliance issues related to movement of the device from one attachment site to another. However, our supplementary analyses, showing no mean difference between changes in wrist- and waist-worn accelerometer-determined sleep duration, suggest waist-worn accelerometry to present a reasonable estimate of change in sleep duration during the intervention.

Since the present analyses were exploratory, we did not correct for multiple testing which increased the risk of findings by chance. The present analyses were based on data from a larger intervention study, and power calculations and determination of sample size were not based on sleep parameters. Thus, the study might not have been sufficiently powered to study the effects of exercise on some of the sleep parameters examined. Therefore, our observations should be verified in future randomized controlled trials powered to detect effects of different types of exercise on sleep. Furthermore, the greater than expected drop-out rates may increase the risk of bias and affect the generalizability of the results in respect to evaluating the potential effects of exercise on sleep. Also, the greater than expected drop-out during the intervention stresses the challenges related to implementation and maintenance of increased physical activity levels in an everyday life perspective. Moreover, the narrow inclusion criteria in the present study limit the generalizability of our findings. Although imbalances in sleep characteristics across groups at baseline are assumed to be present at random, we cannot exclude that this might have affected some of the findings. Strengths of the study include the excellent adherence to the exercise regimen and the use of objective measurements as well as the high number of days with valid sleep data during the assessment periods, which may have reduced the potential impact of inherent measurement errors [44].

In summary, the present study suggests that effects of exercise on sleep may be restricted to leisure-time exercise with a short-term effect on sleep duration after vigorous intensity exercise, whereas sleep duration variability was lower after moderate intensity exercise and numerically lower after vigorous intensity exercise after 6 months, suggesting a more regular sleep pattern compared to physically inactive controls. However, the effects were primarily driven by shorter and more irregular sleep over time in the control group which affects interpretation of the findings. Our findings should be verified in future randomized controlled trials powered to detect effects of different types of exercise on sleep. Furthermore, future studies should investigate effects of different modalities of exercise on objectively measured sleep in individuals with poor sleep and greater degrees of adiposity.

## Figures and Tables

**Table 1:** Baseline characteristics of all randomized participants and the participants included in the *per protocol* analyses.

Characteristics	All randomized	CON	BIKE	MOD	VIG
Number of participants	130	16	22	30	24
Age (years)	34 (7)	35 (7)	35 (7)	32 (7)	37 (7)
Gender (women/men)	68/62	7/9	12/10	15/15	13/11
Anthropometric variables					
Body weight (kg)	89.9 (12.5)	93.2 (11.8)	90.2 (12.5)	89.7 (11.0)	91.2 (13.1)
Body mass index (kg/m^2^)	29.6 (2.6)	30.1 (2.3)	30.1 (3.3)	29.3 (2.0)	29.8 (2.3)
Fat (%)	39.0 (7.5)	39.0 (6.8)	38.4 (8.3)	37.8 (7.8)	38.8 (6.6)
Waist circumference (cm)	94.0 (1.0)	97.5 (8.3)	94.6 (10.8)	93.0 (9.0)	94.7 (9.4)
Android fat (%)	48.8 (6.2)	49.2 (5.6)	48.8 (6.7)	48.1 (6.5)	48.8 (5.0)
Exercise test variables					
VO_2_peak (ml/kg/min)	29.1 (4.9)	29.3 (6.5)	29.8 (4.5)	29.5 (4.5)	28.9 (4.8)
Maximal heart rate (bpm)	184 (10.9)	186 (14)	185 (11)	185 (8)	182 (12)

Data are shown as mean (standard deviation). BIKE, active commuting group; bpm, beats per minute; CON, control group; MOD, moderate intensity exercise group; VIG, vigorous intensity exercise group; VO_2_, oxygen uptake.

**Table 2 tab2:** Training compliance of the participants included in the *per protocol* analyses.

Training compliance	*n*	BIKE	*n*	MOD	*n*	VIG
Exercise frequency (sessions/week)^a^						
0–3 months	22	3.9 (0.6)	30	4.0 (0.5)	24	4.1 (0.5)
0–6 months	19	3.9 (0.4)	28	3.9 (0.5)	23	3.9 (0.6)
Training EE (kcal/session)^b^						
(i) Women						
0–3 months	12	324 (286; 344)	15	334 (294; 363)	13	300 (275; 334)
0–6 months	11	322 (315; 355)	13	318 (295; 354)	12	307 (281; 323)
(ii) Men						
0–3 months	10	441 (421; 493)	15	446 (384; 511)	11	409 (306; 475)
0–6 months	8	473 (447; 507)	15	471 (384; 504)	11	393 (362; 499)
Exercise duration (min/session)^a^						
0–3 months	22	45 (10)	30	52 (11)^†^	24	36 (7)^†,$^
0–6 months	19	46 (10)	28	54 (11)^†^	23	37 (8)^†,$^
Total training EE (kcal)^b^						
0–3 months	22	19,340 (17,660; 21,616)	30	19,715 (16,015; 22,969)	24	18,198 (15,984; 22,215)
0–6 months	19	37,609 (33,750; 48,682)	28	42,457 (32,577; 49,359)	23	37,663 (33,507; 43,681)
% prescribed kcal^a^						
0–3 months	22	94 (22)	30	103 (20)	24	93 (16)
0–6 months	19	95 (12)	28	98 (18)	23	90 (15)
Exercise intensity (%VO_2_peak reserve)^a^						
0–3 months	22	54 (8)	30	50 (7)	24	68 (8)^†,$^
0–6 months	19	54 (8)	28	49 (6)^†^	23	65 (6)^†,$^
Distance (km/day)^b^						
(i) Women						
0–3 months	12	11.7 (8.7; 14.7)	—	—	—	—
0–6 months	10	11.6 (10.6; 14.5)	—	—	—	—
(ii) Men						
0–3 months	11	15.2 (12.7; 18.8)	—	—	—	—
0–6 months	8	15.7 (13.7; 17.6)	—	—	—	—

Data are shown as ^a^mean (standard deviation) and ^b^median (interquartile range). ^†^ *p* < 0.05 vs. BIKE; ^$^ *p* < 0.05 vs. MOD. BIKE, active commuting group; EE, energy expenditure; MOD, moderate intensity exercise group; VIG, vigorous intensity exercise group; VO_2_, oxygen uptake.

**Table 3:** Summary of raw sleep data at baseline, 3 months, and 6 months of the participants included in the *per protocol* analyses.

Characteristics	*n*	CON	*n*	BIKE	*n*	MOD	*n*	VIG
Accelerometry								
Sleep duration (min/night)^a^								
Baseline	12	414 (47)	20	410 (52)	24	417 (42)	24	427 (41)
3 months	12	393 (57)	20	394 (44)	24	418 (40)	24	429 (39)
Baseline	13	414 (42)	17	404 (47)	26	420 (41)	22	427 (40)
6 months	13	421 (54)	17	407 (47)	26	414 (46)	22	419 (51)
Sleep duration variability (min/night)^b^								
Baseline	12	60 (37; 80)	20	46 (39; 58)	24	50 (39; 70)	24	52 (36; 63)
3 months	12	66 (42; 71)	20	56 (47; 74)	24	50 (36; 66)	24	55 (39; 71)
Baseline	13	41 (34; 75)	17	49 (39; 62)	26	50 (38; 70)	22	52 (34; 64)
6 months	13	60 (46; 77)	17	42 (33; 66)	26	43 (32; 58)	22	47 (29; 67)
Sleep midpoint variability (min/night)^b^								
Baseline	12	50 (38; 59)	20	30 (24; 54)	24	40 (27; 60)	24	37 (24; 45)
3 months	12	39 (31; 63)	20	44 (33; 63)	24	49 (26; 67)	24	43 (34; 52)
Baseline	13	50 (38; 53)	17	29 (23; 55)	26	44 (30; 59)	22	37 (26; 45)
6 months	13	45 (39; 53)	17	40 (35; 47)	26	36 (27; 51)	22	38 (27; 45)
Weekday sleep duration (min/night)^a^								
Baseline	12	411 (48)	20	406 (57)	24	407 (51)	24	420 (53)
3 months	12	384 (56)	20	392 (55)	24	419 (52)	24	418 (45)
Baseline	13	408 (48)	17	398 (57)	26	410 (50)	22	419 (54)
6 months	13	413 (63)	17	403 (47)	26	414 (49)	22	419 (66)
Weekend sleep duration (min/night)^a^								
Baseline	12	430 (96)	20	415 (71)	24	445 (63)	24	447 (51)
3 months	12	417 (83)	20	398 (57)	24	415 (59)	24	458 (88)
Baseline	13	433 (76)	17	413 (70)	26	447 (63)	22	446 (44)
6 months	13	441 (79)	17	418 (66)	26	414 (66)	22	421 (57)
Number of nights^b^								
Baseline	12	7 (7; 7)	20	7 (7; 7)	24	7 (7; 7)	24	7 (6; 7)
3 months	12	7 (7; 7)	20	7 (7; 7)	24	7 (7; 7)	24	7 (6; 7)
Baseline	13	7 (7; 7)	17	7 (7; 7)	26	7 (7; 7)	22	7 (6; 7)
6 months	13	7 (7; 7)	17	7 (7; 7)	26	7 (7; 7)	22	7 (6; 7)
Sleep logs								
Bedtime (hh:mm)^b^								
Baseline	12	23:33 (22:57; 00:10)	18	23:00 (22:43; 23:31)	22	23:23 (22:38; 00:14)	23	23:00 (22:30; 23:30)
3 months	12	23:27 (23:00; 23:50)	18	23:27 (22:41; 00:08)	22	23:28 (23:40; 23:53)	23	23:00 (22:35; 23:50)
Baseline	13	23:40 (22:59; 00:09)	16	23:00 (22:40; 23:32)	24	23:08 (22:30; 00:00)	22	23:00 (22:33; 23:30)
6 months	13	23:10 (23:00; 00:00)	16	23:20 (22:38; 00:09)	24	23:00 (22:30; 23:53)	22	23:00 (22:19; 23:34)
Time to sleep (hh:mm)^b^								
Baseline	12	23:40 (22:34; 00:30)	18	23:43 (23:13; 00:14)	22	23:55 (23:18; 00:35)	23	23:35 (22:50; 00:00)
3 months	12	23:47 (23:17; 00:10)	18	23:59 (22:53; 00:45)	22	00:00 (23:17; 00:30)	23	23:40 (23:10; 00:13)
Baseline	13	23:50 (22:57; 00:34)	16	23:43 (23:10; 00:18)	24	23:40 (23:20; 00:30)	22	23:34 (22:50; 23:55)
6 months	13	00:00 (23:27; 00:18)	16	23:45 (23:20; 00:35)	24	23:40 (23:15; 00:26)	22	23:33 (22:53; 00:15)
Wake-up time (hh:mm)^b^								
Baseline	12	06:00 (05:45; 08:30)	18	06:46 (06:00; 07:30)	22	07:16 (06:05; 08:13)	23	06:40 (06:10; 07:30)
3 months	12	06:18 (05:44; 07:30)	18	06:45 (06:15; 08:10)	22	07:00 (05:58; 08:00)	23	06:30 (06:15; 07:40)
Baseline	13	06:30 (05:45; 07:58)	16	06:33 (05:55; 07:10)	24	07:05 (06:00; 08:10)	22	06:31 (06:05; 07:30)
6 months	13	06:45 (06:08; 07:55)	16	06:30 (06:18; 07:30)	24	06:53 (06:00; 07:30)	22	06:22 (06:00; 07:00)
Questionnaires								
Sleep quality (PSQI total score)^b^								
Baseline	14	4 (3; 7)	18	5 (4; 7)	30	5 (4; 7)	24	6 (3; 7)
3 months	14	4 (3; 5)	18	5 (4; 8)	30	5 (3; 8)	24	4 (3; 6)
Baseline	16	4 (3; 8)	18	5 (4; 7)	28	5 (4; 7)	23	6 (3; 7)
6 months	16	6 (3; 8)	18	6 (4; 8)	28	6 (3; 8)	23	5 (3; 7)
Sleepiness (ESS total score)^a^								
Baseline	14	7 (3)	16	7 (4)	28	9 (3)	23	7 (4)
3 months	14	8 (4)	16	8 (4)	28	8 (4)	23	8 (4)
Baseline	15	7 (3)	17	7 (4)	27	8 (3)	22	7 (4)
6 months	15	8 (3)	17	6 (4)	27	8 (4)	22	7 (4)

The raw data are shown as ^a^unadjusted mean (standard deviation) and ^b^median (interquartile range). Sleep duration variability and sleep midpoint variability were calculated as the sum of the absolute differences between the mean and each night of measurement divided by the number of nights measured. Sleep logs: the median of each assessment period for each participant was calculated and the data presented are the medians (interquartile ranges) of these observations. BIKE, active commuting group; CON, control group; ESS, Epworth Sleepiness Scale; MOD, moderate intensity exercise group; PSQI, Pittsburgh Sleep Quality Index; VIG, vigorous intensity exercise group.

**Table 4:** Estimated changes in sleep characteristics of the participants included in the *per protocol* analyses.

	CON		BIKE				MOD				VIG				*p* BIKE vs. MOD	*p* BIKE vs. VIG	*p* MOD vs. VIG
	*n*	Within group change	*n*	Within group change	Change vs. CON	*p* vs. CON	*n*	Within group change	Change vs. CON	*p* vs. CON	*n*	Within group change	Change vs. CON	*p* vs. CON			
Accelerometry																	
Sleep duration (min)																	
0–3 months	12	−22 (−44; −1)	20	−20 (−37; −3)	2 (−24; 29)	0.85	24	−1 (−17; 15)	21 (−5; 47)	0.11	24	8 (−9; 23)	29 (3; 55)	0.03	0.10	0.02	0.45
0–6 months	13	6 (−17; 30)	17	−3 (−24; 18)	−9 (−40; 21)	0.54	26	−7 (−24; 10)	−13 (−42; 15)	0.36	22	−2 (−20; 16)	−8 (−38; 21)	0.57	0.77	0.93	0.69
Sleep duration variability (%)																	
0–3 months	12	10 (−14; 40)	20	20 (−1; 45)	9 (−19; 45)	0.56	24	−4 (−20; 14)	−13 (−34; 16)	0.35	24	11 (−7; 32)	1 (−24; 35)	0.93	0.08	0.55	0.22
0–6 months	13	20 (−9; 59)	17	−6 (−27; 20)	−22 (−45; 12)	0.17	26	−17 (−32; 3)	−31 (−50; −3)	0.03	22	−14 (−31; 8)	−28 (−49; 1)	0.06	0.44	0.59	0.82
Sleep midpoint variability (%)																	
0–3 months	12	10 (−18; 48)	20	30 (3; 64)	18 (−18; 71)	0.37	24	15 (−7; 43)	5 (−26; 50)	0.78	24	16 (−6; 44)	5 (−26; 51)	0.77	0.44	0.46	0.98
0–6 months	13	10 (−17; 46)	17	6 (−17; 35)	−4 (−33; 37)	0.82	26	−15 (−30; 5)	−23 (−44; 8)	0.13	22	0 (−20; 24)	−10 (−36; 27)	0.56	0.16	0.71	0.28
Weekday sleep duration (min)																	
0–3 months	12	−27 (−52; −2)	20	−17 (−36; 3)	10 (−21; 41)	0.52	24	8 (−10; 26)	34 (4; 64)	0.03	24	4 (−14; 22)	31 (0; 61)	0.047	0.06	0.12	0.76
0–6 months	13	7 (−21; 34)	17	−1 (−24; 23)	−7 (−43; 28)	0.69	26	1 (−18; 21)	−5 (−38; 27)	0.75	22	7 (−14; 28)	1 (−33; 34)	0.98	0.90	0.62	0.68
Weekend sleep duration (min)																	
0–3 months	12	−17 (−60; 26)	20	−34 (−67; 1)	−17 (−69; 36)	0.53	24	−23 (−55; 9)	−6 (−57; 45)	0.81	24	20 (−11; 52)	37 (−14; 88)	0.15	0.63	0.02	0.04
0–6 months	13	6 (−31; 44)	17	−15 (−48; 18)	−21 (−69; 27)	0.38	26	−26 (−53; 2)	−32 (−76; 12)	0.16	22	−17 (−47; 13)	−23 (−69: 22)	0.31	0.61	0.93	0.65
Sleep quality																	
PSQI total score (%)																	
0–3 months	14	−15 (−33; 7)	18	1 (−18; 25)	19 (−12; 61)	0.25	30	−3 (−17; 15)	15 (−13; 51)	0.33	24	−22 (−35; −6)	−8 (−31; 23)	0.57	0.76	0.06	0.07
0–6 months	16	8 (−16; 39)	18	7 (−15; 36)	−1 (−29; 39)	0.95	28	−7 (−23; 13)	−14 (−37; 17)	0.34	23	−16 (−32; 4)	−22 (−44; 7)	0.12	0.35	0.13	0.47
Sleepiness																	
ESS total score																	
0–3 months	14	0.9 (−0.4; 2.2)	16	1.1 (−0.1; 2.3)	0.2 (−1.6; 2.0)	0.82	28	−0.1 (−1.1; 0.8)	−1.0 (−2.6; 0.6)	0.20	23	0.9 (−0.1; 2.0)	0 (−1.6; 1.7)	0.97	0.11	0.84	0.13
0–6 months	15	0.6 (−0.7; 1.9)	17	−0.9 (−2.1; 0.3)	−1.5 (−3.2; 0.2)	0.08	27	−0.6 (−1.5; 0.3)	−1.2 (−2.8; 0.3)	0.12	23	−0.5 (−1.5; 0.6)	−1.1 (−2.7; 0.5)	0.19	0.67	0.57	0.85

Data are estimated mean changes (95% CI) for parametric data and percentage change (95% CI) for nonparametric data. Changes from baseline to 3 and 6 months were assessed using a mixed model adjusted for gender, age, and baseline variation. The model was baseline constrained, and differences at baseline were thereby ascribed to the individual level. Sleep duration variability and sleep midpoint variability were calculated as the sum of the absolute differences between the mean and each night of measurement divided by the number of nights measured. BIKE, active commuting group; CON, control group; ESS, Epworth Sleepiness Scale; MOD, moderate intensity exercise group; PSQI, Pittsburgh Sleep Quality Index; VIG, vigorous intensity exercise group.

**Table 5:** Associations between changes in sleep characteristics and adiposity in the intervention groups.

	Sleep duration, min (*n* = 67)		Sleep duration variability, min (*n* = 67)		Sleep midpoint variability, min (*n* = 67)		Sleep quality, PSQI total score (*n* = 72)		Sleepiness, ESS total score (*n* = 67)	
	*β* (95% CI)	*p*	*β* (95% CI)	*p*	*β* (95% CI)	*p*	*β* (95% CI)	*p*	*β* (95% CI)	*p*
*Associations between changes in sleep and adiposity from baseline to 3 months*										
Fat (%)^a^	−0.01 (−0.03; 0.00)	0.10	0.01 (−0.02; 0.03)	0.55	−0.02 (−0.03; 0.00)	0.07	0.30 (0.04; 0.57)	0.03	0.31 (0.09; 0.54)	<0.01
WC (cm)	−0.01 (−0.05; 0.03)	0.63	−0.01 (−0.08; 0.05)	0.66	−0.03 (−0.08; 0.01)	0.15	0.77 (0.11; 1.43)	0.02	−0.08 (−0.74; 0.58)	0.82
Android fat (%)^a^	−0.02 (−0.04; 0.01)	0.12	−0.01 (−0.05; 0.02)	0.51	−0.03 (−0.05; −0.01)	0.03	0.22 (−0.16; 0.60)	0.26	0.59 (0.28; 0.90)	<0.001

	Sleep duration, min (*n* = 65)		Sleep duration variability, min (*n* = 65)		Sleep midpoint variability, min (*n* = 65)		Sleep quality, PSQI total score (*n* = 69)		Sleepiness, ESS total score (*n* = 66)	

*Associations between changes in sleep and adiposity from baseline to 6 months*										
Fat (%)^a^	0.00 (−0.02; 0.01)	0.77	−0.01 (−0.05; 0.02)	0.35	−0.03 (−0.06; 0.00)	0.07	−0.05 (−0.32; 0.22)	0.71	0.11 (−0.18; 0.39)	0.46
WC (cm)	−0.02 (−0.06; 0.01)	0.15	0.00 (−0.07; 0.07)	0.90	−0.03 (−0.10; 0.05)	0.44	−0.12 (−0.66; 0.42)	0.66	0.54 (−0.05; 1.13)	0.07
Android fat (%)^a^	−0.01 (−0.03; 0.01)	0.24	−0.02 (−0.06; 0.02)	0.31	−0.04 (−0.08; 0.00)	0.08	0.14 (−0.20; 0.48)	0.41	0.38 (0.04; 0.72)	0.03

Analyses included participants from BIKE, MOD, and VIG who were included in per protocol analyses. Data are shown as unstandardized regression coefficients (*β*) (95% CI). ^a^Presented as percentage points. Sleep duration variability and sleep midpoint variability were calculated as the sum of the absolute differences between the mean and each night of measurement divided by the number of nights measured. Adiposity variables were dependent variables in the multiple regression analyses, which were adjusted for age, gender, specific adiposity and sleep variables at baseline, and intervention group. BIKE, active commuting group; ESS, Epworth Sleepiness Scale; MOD, moderate intensity exercise group; PSQI, Pittsburgh Sleep Quality Index; VIG, vigorous intensity exercise group; WC, waist circumference.

## Data Availability

All requests for data access should be addressed to the corresponding author. Proposals requesting data access will have to specify how it is planned to use the data, and transfer agreement is required by our institution.

## Supplementary Materials

Supplementary Materials Supplementary Table 1: estimated sleep duration using waist and wrist as attachment sites (*n* = 61). Supplementary Figure 1: comparison between changes (mean of first four nights minus mean of last four days) in estimated sleep duration using waist and wrist as attachment sites (*n* = 61). Pearson's correlation coefficient (*r*) = 0.83 (*p* < 0.001).

## References

[1] Schmid S. M., Hallschmid M., Schultes B. (2015). The metabolic burden of sleep loss. *The Lancet Diabetes & Endocrinology*.

[2] Quist J. S., Sjödin A., Chaput J.-P., Hjorth M. F. (2016). Sleep and cardiometabolic risk in children and adolescents. *Sleep Medicine Reviews*.

[3] Knutson K. L., Van Cauter E. (2008). Associations between sleep loss and increased risk of obesity and diabetes. *Annals of the New York Academy of Sciences*.

[4] Driver H. S., Taylor S. R. (2000). Exercise and sleep. *Sleep Medicine Reviews*.

[5] Chennaoui M., Arnal P. J., Sauvet F., Léger D. (2015). Sleep and exercise: a reciprocal issue?. *Sleep Medicine Reviews*.

[6] Kwon A. M., Shin C. (2016). Structural equation modelling for the effect of physical exercise on excessive daytime sleepiness. *Public Health*.

[7] Sherrill D. L., Kotchou K., Quan S. F. (1998). Association of physical activity and human sleep disorders. *Archives of Internal Medicine*.

[8] Morgan K. (2003). Daytime activity and risk factors for late-life insomnia. *Journal of Sleep Research*.

[9] Dishman R. K., Sui X., Church T. S., Kline C. E., Youngstedt S. D., Blair S. N. (2015). Decline in cardiorespiratory fitness and odds of incident sleep complaints. *Medicine & Science in Sports & Exercise*.

[10] Kredlow M. A., Capozzoli M. C., Hearon B. A., Calkins A. W., Otto M. W. (2015). The effects of physical activity on sleep: a meta-analytic review. *Journal of Behavioral Medicine*.

[11] Kelley G. A., Kelley K. S. (2017). Exercise and sleep: a systematic review of previous meta-analyses. *Journal of Evidence-Based Medicine*.

[12] Kjeldsen J. S., Rosenkilde M., Nielsen S. W. (2013). Effect of different doses of exercise on sleep duration, sleep efficiency and sleep quality in sedentary, overweight men. *Bioenergetics: Open Access*.

[13] Buman M. P., King A. C. (2010). Exercise as a treatment to enhance sleep. *American Journal of Lifestyle Medicine*.

[14] Rosenkilde M., Petersen M. B., Gram A. S. (2017). The GO-ACTIWE randomized controlled trial—an interdisciplinary study designed to investigate the health effects of active commuting and leisure time physical activity. *Contemporary Clinical Trials*.

[15] Quist J. S., Rosenkilde M., Petersen M. B., Gram A. S., Sjödin A., Stallknecht B. (2018). Effects of active commuting and leisure-time exercise on fat loss in women and men with overweight and obesity: a randomized controlled trial. *International Journal of Obesity*.

[16] Quist J. S., Blond M. B., Gram A. S Effects of active commuting and leisure-time exercise on appetite in individuals with overweight and obesity. *Journal of Applied Physiology*.

[17] Gram A. S., Bladbjerg E.-M., Quist J. S., Petersen M. B., Rosenkilde M., Stallknecht B. (2017). Anti-inflammatory effects of active commuting and leisure time exercise in overweight and obese women and men: a randomized controlled trial. *Atherosclerosis*.

[18] Gram A. S., Petersen M. B., Quist J. S., Rosenkilde M., Stallknecht B., Bladbjerg E. M. (2018). Effects of 6 Months of active commuting and leisure-time exercise on fibrin turnover in sedentary individuals with overweight and obesity: a randomised controlled trial. *Journal of Obesity*.

[19] Nordby P., Auerbach P. L., Rosenkilde M. (2012). Endurance training per se increases metabolic health in young, moderately overweight men. *Obesity*.

[20] Rosenkilde M., Auerbach P., Reichkendler M. H., Ploug T., Stallknecht B. M., Sjödin A. (2012). Body fat loss and compensatory mechanisms in response to different doses of aerobic exercise—a randomized controlled trial in overweight sedentary males. *American Journal of Physiology-Regulatory, Integrative and Comparative Physiology*.

[21] World Health Organization (2010). *Global Recommendations on Physical Activity for Health*.

[22] Garber C. E., Blissmer B., Deschenes M. R. (2011). Quantity and quality of exercise for developing and maintaining cardiorespiratory, musculoskeletal, and neuromotor fitness in apparently healthy adults. *Medicine & Science in Sports & Exercise*.

[23] Cole R. J., Kripke D. F., Gruen W., Mullaney D. J., Gillin J. C. (1992). Automatic sleep/wake identification from wrist activity. *Sleep*.

[24] Hjorth M. F., Chaput J.-P., Damsgaard C. T. (2012). Measure of sleep and physical activity by a single accelerometer: can a waist-worn actigraph adequately measure sleep in children?. *Sleep and Biological Rhythms*.

[25] Kjeldsen J. S., Hjorth M. F., Andersen R. (2014). Short sleep duration and large variability in sleep duration are independently associated with dietary risk factors for obesity in Danish school children. *International Journal of Obesity*.

[26] Buysse D. J., Reynolds C. F., Monk T. H., Berman S. R., Kupfer D. J. (1989). The Pittsburgh sleep quality index: a new instrument for psychiatric practice and research. *Psychiatry Research*.

[27] Johns M. W. (1991). A new method for measuring daytime sleepiness: the epworth sleepiness scale. *Sleep*.

[28] Mendelson M., Borowik A., Michallet A.-S. (2016). Sleep quality, sleep duration and physical activity in obese adolescents: effects of exercise training. *Pediatric Obesity*.

[29] Lastella M., Roach G. D., Halson S. L., Sargent C. (2015). Sleep/wake behaviours of elite athletes from individual and team sports. *European Journal of Sport Science*.

[30] Halson S. L., Juliff L. E. (2017). Sleep, sport, and the brain. *Progress in Brain Research*.

[31] Yetish G., Kaplan H., Gurven M. (2015). Natural sleep and its seasonal variations in three pre-industrial societies. *Current Biology*.

[32] Lehnkering H., Siegmund R. (2007). Influence of chronotype, season, and sex of subject on sleep behavior of young adults. *Chronobiology International*.

[33] He F., Bixler E. O., Liao J. (2015). Habitual sleep variability, mediated by nutrition intake, is associated with abdominal obesity in adolescents. *Sleep Medicine*.

[34] McHill A. W., Wright K. P. (2017). Role of sleep and circadian disruption on energy expenditure and in metabolic predisposition to human obesity and metabolic disease. *Obesity Reviews*.

[35] Pereira É. F., Moreno C., Louzada F. M. (2014). Increased commuting to school time reduces sleep duration in adolescents. *Chronobiology International*.

[36] Kline C. E., Sui X., Hall M. H. (2012). Dose-response effects of exercise training on the subjective sleep quality of postmenopausal women: exploratory analyses of a randomised controlled trial. *BMJ Open*.

[37] Yang P.-Y., Ho K.-H., Chen H.-C., Chien M.-Y. (2012). Exercise training improves sleep quality in middle-aged and older adults with sleep problems: a systematic review. *Journal of Physiotherapy*.

[38] Youngstedt S. D. (2003). Ceiling and floor effects in sleep research. *Sleep Medicine Reviews*.

[39] Ng W. L., Stevenson C. E., Wong E. (2017). Does intentional weight loss improve daytime sleepiness? A systematic review and meta-analysis. *Obesity Reviews*.

[40] Pearson N., Biddle S. J. H. (2011). Sedentary behavior and dietary intake in children, adolescents, and adults. *American Journal of Preventive Medicine*.

[41] Young T., Peppard P. E., Taheri S. (2005). Excess weight and sleep-disordered breathing. *Journal of Applied Physiology*.

[42] Thomasouli M.-A., Brady E. M., Davies M. J. (2013). The impact of diet and lifestyle management strategies for obstructive sleep apnoea in adults: a systematic review and meta-analysis of randomised controlled trials. *Sleep and Breathing*.

[43] Araghi M. H., Chen Y.-F., Jagielski A. (2013). Effectiveness of lifestyle interventions on obstructive sleep apnea (OSA): systematic review and meta-analysis. *Sleep*.

[44] Sadeh A. (2011). The role and validity of actigraphy in sleep medicine: an update. *Sleep Medicine Reviews*.

