# Supplementary material for: Effects of Exercise Domain and Intensity on Sleep in Women and Men with Overweight and Obesity
Source: J Obes. 2019 Apr 7;2019:2189034. doi: 10.1155/2019/2189034 (PMC6476111; doi:10.1155/2019/2189034)
Supplement: Supplementary Materials — Supplementary Table 1: estimated sleep duration using waist and wrist as attachment sites (n = 61). Supplementary Figure 1: comparison between changes (mean of first four nights minus mean of last four days) in estimated sleep duration using waist and wrist as attachment sites (n = 61). Pearson's correlation coefficient (r) = 0.83 (p < 0.001). [file 2189034.f1.docx]

| **Supplementary Table 1**: Estimated sleep duration using waist and wrist as attachment sites (n=61) | | | | | | |
| --- | --- | --- | --- | --- | --- | --- |
|  | Waist | Wrist | ∆ | P-value^1^ | r | P-value^2^ |
| Day 1 | 540±50 | 470±50 | 70±26 | <0.001 | 0.86 | <0.001 |
| Day 2 | 542±55 | 471±64 | 71±48 | <0.001 | 0.68 | <0.001 |
| Day 3 | 514±67 | 438±72 | 76±41 | <0.001 | 0.83 | <0.001 |
| Day 4 | 551±40 | 474±40 | 77±31 | <0.001 | 0.69 | <0.001 |
| Day 5 | 547±48 | 473±47 | 74±37 | <0.001 | 0.70 | <0.001 |
| Day 6 | 543±37 | 469±44 | 74±32 | <0.001 | 0.70 | <0.001 |
| Day 7 | 551±39 | 473±47 | 78±31 | <0.001 | 0.75 | <0.001 |
| Day 8 | 557±45 | 483±52 | 74±32 | <0.001 | 0.79 | <0.001 |
| Mean of day 1 to 4 | 537±35 | 463±40 | 74±25 | <0.001 | 0.78 | <0.001 |
| Mean of day 5 to 8 | 550±32 | 474±35 | 75±26 | <0.001 | 0.69 | <0.001 |
| ∆(mean of day 1 to 4) - (mean of day 5 to 8) | -12.6±28 | -11.1±33 | **-1.5±19^3^** | **0.52** | **0.83^4^** | **<0.001** |
| Sleep duration (min/night) is reported as mean ± standard deviation using the algorithm by Sadeh.  ^1^Testing mean difference between the use of waist and wrist as attachment site by paired t-test  ^2^Testing significance of Pearson’s correlation coefficients (r) between the use of waist and wrist as attachment site  ^3^95%CI: -6.3 to 3.2 | | | | | | |


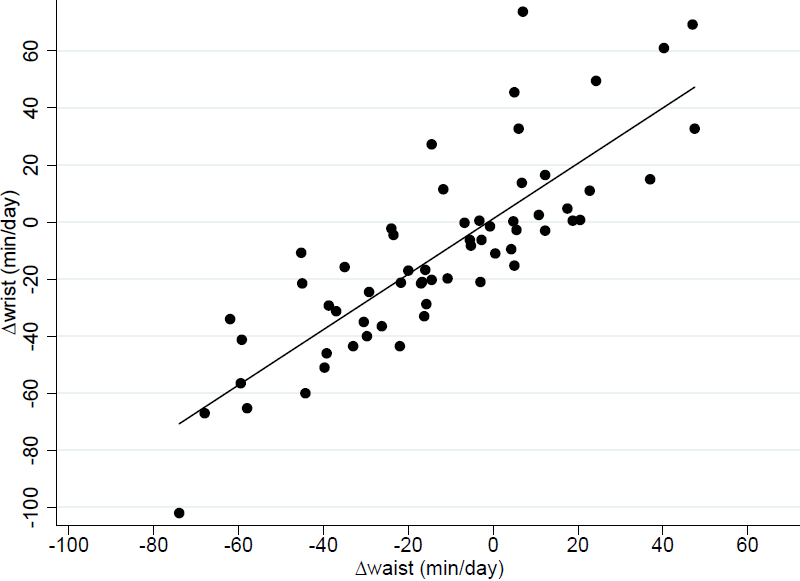


**Supplementary Figure 1**: Comparison between changes (mean of first four nights minus mean of last four days) in estimated sleep duration using waist and wrist as attachment site (n=61).

Pearson’s correlation coefficient (r)=0.83 (P<0.001).
